# Size-fractionated carbonaceous and iron-rich particulate matter in urban environments of France and Senegal

**DOI:** 10.1007/s11356-024-35729-x

**Published:** 2024-12-19

**Authors:** Laurence Delville, Jean-François Léon, Mélina Macouin, Yann-Philippe Tastevin, François Demory, Arnaud Proietti, Pedro Henrique da Silva Chibane, Maria Dias Alves, Mayoro Gueye, Laure Laffont, Eric Gardrat, Sonia Rousse, Loic Drigo, Andréa Teixeira Ustra

**Affiliations:** 1 Laboratoire d’Aérologie, Université de Toulouse, CNRS, IRD, UPS, Toulouse, France; 2 Géosciences Environnement Toulouse, Université de Toulouse, CNES, CNRS, IRD, UPS, Toulouse, France; 3 ESS - Unité Mixte Internationale “Environnement Santé Sociétés”, CNRS, Université Cheikh Anta Diop de Dakar, Dakar, Senegal; 4 Centre Européen de Recherche et d’Enseignement des Géosciences de l’Environnement, Aix-Marseille Université, CNRS, IRD, INRAE, Aix-en-Provence, France; 5 Centre de micro-caractérisation Raimond Castaing, Université de Toulouse, CNRS, INP Toulouse, INSA Toulouse, UFTMP, UPS, Toulouse, France; 6 Instituto de Astronomia, Geofísica e Ciências Atmosféricas, Universidade de São Paulo, USP, São Paulo, Brazil

**Keywords:** Urban pollution, Carbonaceous aerosols, Iron-rich particles, Magnetism, Size distribution, Western Africa

## Abstract

Road traffic is one of the main sources of particulate matter in the urban environment, emitting particulate organic and elemental carbon compounds and metal-rich particles through combustion and brakes and tires wear. In Western Africa, the carbon and metal composition of airborne particles is also influenced by additional sources linked to biomass combustion and recent industrialization. Here, we investigated the impact of combustion-related and non-combustion-related emissions on the distribution of carbonaceous fractions and iron-rich particles in two urban environments in France and Senegal. The supermicron fraction ($D_a>{1}$µm) showed a significantly higher isothermal remanent magnetization (SIRM) than finer fractions, accounting for 79% in France and 81% in Senegal of the total SIRM. In the submicron fraction ($D_a<{1}$µm), we noted significantly higher concentrations of total carbon (TC) and elemental carbon (EC) than for other fractions, both accounting for 71% in France and 68% and 75% in Senegal of the total and elemental particulate carbon concentration, respectively. Electron microscope observations revealed the presence of iron-rich particles for $D_a<{0.2}$µm, however, associated with a weak SIRM. Such iron particles may be produced by combustion or abrasion while we suspect that emissions by the abrasion process produce larger particles.

**Supplementary Information:**

The online version contains supplementary material available at 10.1007/s11356-024-35729-x.

## Introduction

The harmful effects of particulate matter (PM) on human health have been largely demonstrated by epidemiological studies (Liu et al. 2023; Suryadhi et al. 2020; Ferreira et al. 2022; Leikauf et al. 2020; Chan et al. 2019). Among the 7.3 billion people directly exposed to unsafe average annual PM2.5 (PM having an aerodynamic diameter less than 2.5µm) concentrations, 80% live in low- and middle-income countries (Rentschler and Leonova 2023).

The size and chemical composition of airborne particles varies widely throughout the world (McDuffie et al. 2020) due to the diversity of emission sources, chemical transformation, and atmospheric transport processes (Seinfeld and Pandis 2006). Soot carbon and PM10 (PM having an aerodynamic diameter less than 10µm) emissions have been increasing in Africa since the 90s mainly due to the increasing use of fossil fuels for road traffic and industrialization (McDuffie et al. 2020; Keita et al. 2021; Crippa et al. 2021). Doumbia et al. (2023) have shown that road traffic emissions in Dakar, Senegal, account for 49% of fine particle ($D_a$ between 1 and 0.2µm) emissions. On the contrary, PM10 emissions in Europe are stable or even decreasing according to global emission inventories (McDuffie et al. 2020; Crippa et al. 2021). Since the 2000s, traffic-related exhaust emissions have been decreasing in Europe mainly due to the reinforcement of regulations on diesel emission and the introduction of electric vehicles (Harrison et al. 2021). The contribution of road traffic emission was estimated to 14% of PM2.5 mass in Paris in 2010 (Bressi et al. 2014).

Road traffic PM has a primary and secondary nature. Tailpipe aerosols are composed of elemental carbon (EC) and organic carbon (OC) with significant amounts of inorganic species (Fraser et al. 1998) and trace metals. Carbonaceous aerosols (EC and OC) are emitted by the incomplete combustion of carbon-containing fuels. Trechera et al. (2023) showed that the concentration number of carbonaceous particles from traffic is preferentially in the fraction below 0.2µm. EC is a good tracer of particulate matter emitted by combustion, such as the combustion of fuel in vehicular internal combustion engines (Pant and Harrison 2013; El Haddad et al. 2009; Patel et al. 2009). EC is also released into the atmosphere by many other anthropogenic activities including industrial manufacturing (Chow et al. 2011; Streets et al. 2004), and biomass and waste burning (Liousse et al. 2010; Chow et al. 2011; Bressi et al. 2014). Bressi et al. (2014) showed that road traffic in Paris is responsible for 45% of EC total emissions. According to Kwon et al. (2020), traffic accounts for 34% of total ultrafine particle emissions in Europe.

Non-exhaust aerosols have a higher content of trace metals (e.g., Fe, Cu, Mn, Sb) than tailpipe emissions. Currently, non-exhaust emissions account for 50% of total traffic-related emissions in Europe (Harrison et al. 2021). Beddows et al. (2023) have recently proposed that vehicle brake wear is a major emission source of coarse magnetic minerals ($D_a>{2.5}$µm) in the UK. The concentration of magnetic minerals in the atmosphere is due to the emission of iron-containing particulate matter. Road traffic is a major contributor to atmospheric Fe, reaching 75% in Paris (Bressi et al. 2014). Magnetic minerals are emitted by brake pads and tyre wear abrasion, resuspension of road dust (Mitchell and Maher 2009; Singh and Kaushik 2021; Pant and Harrison 2013; Piscitello et al. 2021; Jeong et al. 2019), steel industry (Flament et al. 2008), and wood burning (Leite et al. 2021).

Leite et al. (2021) showed an excellent correlation between EC concentrations and saturation isothermal remanent magnetization (SIRM) for specific urban sites in western Africa, suggesting that PM2.5 magnetic parameters are linked to primary particulate emission from combustion sources. However, multiple sources, such as industry and non-exhaust traffic emissions contribute to iron-bearing magnetic particles. We assumed that the size distribution of EC and magnetic mineral concentrations provide us with new information on PM sources. Here, we study the size-segregated concentrations of total particulate carbon (TC) and its elemental and organic fractions along with magnetic mineral concentration. We investigated two different urban environments in France (Toulouse) and in Senegal (Sebikotane). The specific sources in Senegal, namely traffic, industrial, and wood burning were also targeted. Aerosols were sampled on a cascade impactor from PM10 to PM0.2 (PM having an aerodynamic diameter less than 0.2µm) and then analyzed by thermo-optical analysis and isothermal-induced magnetization acquisition. We then carried out scanning electron microscopy (SEM) particle observations to study the morphology and composition of particles and to distinguish the presence of iron-bearing particles in the quasi-ultrafine fraction ($D_a<{0.2}$µm). In order to determine the nature of the magnetic mineral species, we acquired hysteresis and magnetic parameters.

## Methods

### Location and sets of filters collection

Toulouse (N $43^{\circ }36'16''$’, E $1^{\circ }26'38''$) is a medium-sized city in southwest France with a population of 500,000 in 2023. The city is surrounded by a ring road with heavy traffic of around 120,000 vehicles a day. The entire conurbation covers an area of ${460}\text {km}^{2}$ and has a population of around 1,000,000. The sampling site is located 650 m from the ring road, at Paul Sabatier University, on a roof of one-story building surrounded by an open parking lot. A total of 4 sets of filters (Table A1) were collected in April, May, October, and November 2022 under cloudy weather. April was characterized by rainy weather in contrast to May.

Sebikotane (N $14^{\circ }44'29''$, W $17^{\circ }8'7''$) is a former rural community of 28,000 inhabitants, now part of the Dakar-Diamniadio conurbation in Senegal. Sebikotane is cut in two by a busy road, the Route Nationale 2 (N2). N2 is Senegal’s most important interurban road, with traffic estimated at 12,000 vehicles a day in 2012 (Lombard 2015). Sandy tracks link residential neighborhoods to N2. Sebikotane is also home to industries: a used lead acid batteries recycling industry, and two secondary steel industries.

A total of 4 sets of filters (Table A1) were collected in Senegal between the 30^th^ January and the 3^rd^ February 2023. The urban background is a site located outside the Diamniadio pediatric hospital, at a distance of about 140 m from N2 road. The industrial site (hereinafter *industrial*) is located 500 m from the metallurgical industry and 50 m from the N2 road. Sampling at the *industrial* site was carried out during the night and the following morning to minimize the impact of traffic. The traffic site (*traffic*) is at the kerbside, located 2 m from N2 road. The wood-burning site is a wood-fired cooking street restaurant (*wood_burning*) by the N2 road.

The comparison of data between France and Senegal was conducted solely between two urban background sites with a specific focus on studying the impact of location rather than seasonal variations. The primary objective of our study was to analyze the influence of location on PM size distribution, which explains why we have not illustrated a temporal trend of PM size distribution in France in our results.

### Mass distribution

A cascade impactor was used to segregate particles by size (e.g., Mirante et al. 2014; Jaffrezo et al. 2005; Alves et al. 2015; Cesari et al. 2020; Singh and Kaushik 2021; Krudysz et al. 2008; Li et al. 2023; Martins et al. 2020; Beddows et al. 2023). The Dekati Gravimetric Impactor (DGI, Dekati Ltd., Tampere, Finland) is a 4 stages (size fractions) cascade impactor that is operating at a flow rate of 70L.$\min ^{-1}$ (Ruusunen et al. 2011). Particles were impacted on 47mm Whatman quartz microfiber filters and the last backup stage was equipped with a 70mm Whatman quartz microfiber filter (Wang et al. 2015). The classified size ranges are coarse (stage 1, aerodynamic diameter $D_{a}>2.5$ µm), inter-modal (stage 2, $D_a$ between 2.5 and 1µm), accumulation (stage 3, $D_a$ between 1 and 0.5µm), fine (stage 4, $D_a$ between 0.5 and 0.2µm), and quasi-ultrafine (backup filter, $D_{a}<0.2$µm). The duration of sampling varied according to the experimental conditions (Table A1). The volume of air was calculated from the sampling duration and the pomp flow rate. Quartz fiber filters were weighted in an atmospheric controlled room with a Sartorius MC21S microbalance with µg precision (Salma et al. 2020; Djossou et al. 2018; Xu et al. 2019).

### Particulate carbon measurements

The filter weighting and particulate carbon analysis were performed at the LAERO (Toulouse, France). Quartz filters were burned before exposure in an oven at ${550}^{\circ }$C during 36h to reduce their carbon content. All 40 filters were analyzed for particulate carbon concentrations using a thermo-optical analyzer (Lab OC-EC, Sunset Laboratory Inc.). EC and OC deposit concentrations were analyzed on ${0.55}\text {cm}^{2}$ aliquots following the European Supersites for Atmospheric Aerosol Research protocol 2 (Cavalli et al. 2010). Total carbon (TC) is given as the sum of EC and OC. On the first 4 stages of the cascade impactor, we related the particulate carbon content and particle mass analyzed with the thermo-optical analyzer to the number of impacteur spots in the aliquots analyzed for each impaction filter. The OC/EC ratio (Pio et al. 2011) is estimated for each samples.

### Magnetic properties

Thirty-three (33) filters were analyzed at the GET (Toulouse, France) with a spinner magnetometer (AGICO JR-6A). Isothermal remanent magnetizations (IRMs) were obtained after application, of a direct positive field of 1T by a MMPM10 pulse magnetizer. Twenty (20) filters were analyzed in USPMag laboratory at the Universidade de São Paulo (IAG-USP, São Paulo, Brazil) with a DC 2 G Enterprises SQUID Long Core 755R rock magnetometer. IRMs were obtained after the application of a direct positive field of 1T by a 670 IRM Pulse Remanent Magnetizer (2 G Entreprises). The IRM at 1T is considered to be the saturation isothermal remanent magnetization (SIRM). All SIRM values are the mean of two consecutive SIRM$_{1T}$ measurements. The entire 47mm filters were placed in gelcaps. The 70mm filters were cut in half using ceramic scissors before being placed in the gelcaps. SIRM atmospheric signal (${\text {Am}}^{-1}$) was obtained by substracting blank filter values from sampled filter values and normalizing SIRM by air volume. The SIRM mass ratio (A m$^{2}$kg$^{-1}$) provides the magnetic content in PM.

In addition, a vibrating sample magnetometer (VSM 8604, LakeShore) was used at CEREGE (Aix-en-Provence, France) for the characterization of the iron-rich particles. Hysteresis loops were obtained for 25 filters, including 1 set of 5 filters in France (Toulouse, OMP, April 2023) and 4 sets of filters (a total of 20 filters) in Senegal. The following protocol was applied: a maximum field strength of 1.5T, a field step of 10mT, an averaging time of 1s, and 100% vibration. Averaging time and percentage of vibration were chosen to reduce measurement errors due to a low magnetic signal.

All hysteresis loops have been corrected for paramagnetism. Hysteresis parameters, i.e., saturation magnetization (Ms), saturation remanent magnetization (Mrs), and coercivity (Hc), were calculated after correcting hysteresis for the paramagnetic signal. The remanent coercivity (Hcr) was calculated from the back-field DC demagnetization from 1.5T.

To identify the impact of the filter material, we also acquired SIRMs of 8 clean support media commonly used in aerosol research. Blank filters IRMs were acquired with a SQUID 2 G Enterprises DC cryogenic magnetometer.

**Table 1:** (mass) Mass concentration, (TC) total carbon, (EC) elemental carbon, (SIRM) volume and mass normalized saturation isothermal remanent magnetization, and (OC/EC) organic to elemental carbon ratio for each of the impactor stage (s1: $D_a>{2.5}$µm; s2: 1µm$<D_a<{2.5}$µm; s3: 0.5µm$<D_a<{1}$µm; s4: 0.2µm$<D_a<{0.5}$µm) and the backup filter ($D_a<{0.2}$µm) for the samples collected in France and in Senegal

	Location		s1	s2	s3	s4	BckUp
mass	France	Urban	4.3	4.6	3.1	2.0	1.5
µg m$^{-3}$	Senegal	Urban	13.5	10.2	4.9	1.9	8.6
		Industrial	55.2	74.6	20.6	7.4	15.3
		Traffic	71.4	65.9	26.1	11.1	24.3
		Wood burning	182.9	637.3	2808.7	2887.4	480.8
TC	France	Urban	0.3	0.3	0.3	0.4	0.8
µg m$^{-3}$	Senegal	Urban	0.9	0.8	0.8	0.9	1.9
		Industrial	2.8	4.8	3.5	3.2	5.6
		Traffic	3.9	5.2	4.9	5.6	10.3
		Wood burning	83.0	378.7	1901.3	2134.7	285.2
EC	France	Urban	0.1	0.1	0.2	0.2	0.1
µg m$^{-3}$	Senegal	Urban	0.2	0.2	0.3	0.4	0.5
		Industrial	1.4	3.2	2.7	2.6	1.3
		Traffic	0.8	1.6	2.4	3.3	3.9
		Wood burning	12.3	59.7	986.1	995.2	52.0
SIRM	France	Urban	0.7	0.8	0.2	0.1	0.1
$10^{-10}$A m$^{-1}$	Senegal	Urban	7.9	5.2	1.7	0.5	0.8
		Industrial	5.8	5.7	1.4	0.6	NA
		Traffic	17.9	11.9	2.9	0.7	0.1
		Wood burning	7.4	8.2	1.3	1.1	NA
SIRM	France	Urban	1.7	1.8	0.8	0.7	0.6
$10^{-2}$ A m$^{2}$ kg$^{-1}$	Senegal	Urban	6.4	5.5	3.7	2.6	1.8
		Industrial	1.1	0.8	0.7	0.9	NA
		Traffic	2.7	2.0	1.3	0.7	0.1
		Wood burning	0.4	0.1	0.0	0.0	NA
OC/EC	France	Urban	3.1	3.3	1.1	0.9	4.7
	Senegal	Urban	4.6	3.1	1.4	1.2	3.2
		Industrial	1.0	0.5	0.3	0.3	3.3
		Traffic	3.7	2.2	1.1	0.7	1.7
		Wood burning	5.8	5.3	0.9	1.1	4.5

### Microscopic observations

All filters collected at the two urban sites (April sampling in France) and all backup filters at Senegal sources sites were analyzed using a FEG JEOL JSM 7100F TTLS LV scanning electron microscope (SEM) under high-vacuum observation conditions at the Centre Castaing (Toulouse, France). The filters were coated with a layer of nanoscale carbon, to make them electrically conductive, and fixed to the observation plate by a 20nm layer of silver. SEM observations were carried out using the backscattered electron detector (BED) and the secondary electron detector named lower electron detector (LED). Chemical composition was obtained using an Ultim-Max 100 mm^2^ energy dispersive spectroscopy (EDS) system (Oxford Instruments). AZtec software was used for data acquisition and analysis. The EDS acceleration voltage was set to 5 or 10 kV depending on particle size. For the finest particles, 5 kV was used to reduce the interaction volume (pear-shaped) in order to identify only the selected particle. For coarser particles, 10 kV was used.

To assess the presence of zinc- (Zn) and iron-rich (Fe) particles, we analyzed the 5 backup filters using the AZtecFeature tool. We automatically acquired 252 fields of view per filter with a 5% overlap, corresponding to an area of 1mm$^{2}$. We performed an initial fast scan of each field of view with a real acquisition time of 0.1s to discard particles that have atomic percentages in Fe or Zn less than 5wt%. In a second pass (2s per field of view), particles containing at least O 1wt% and Fe 5wt%, or a percentage by weight of Fe greater than the one of Zn, were considered iron-rich particles. Similarly, zinc-rich particles were identified by at least O 1wt% and Zn 5wt%, or a percentage by weight of Zn greater than Fe.

**Fig. 1:**
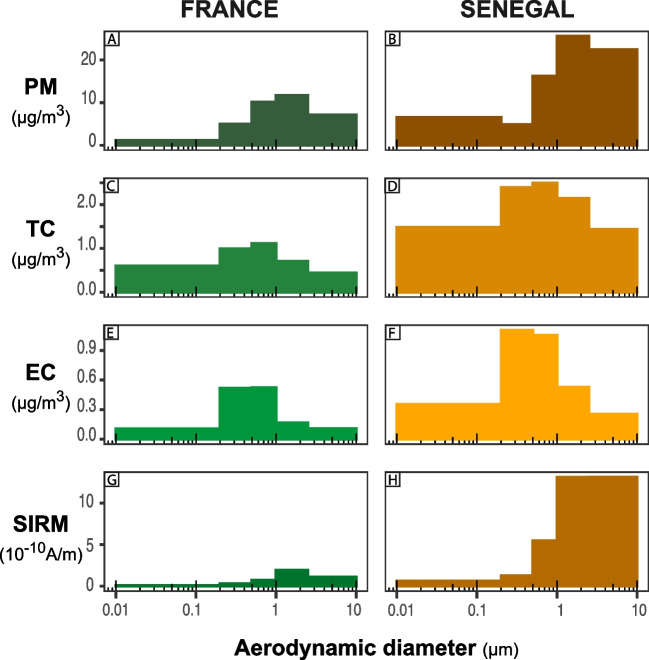
Size-fractionated **A** and **B** particulate matter (PM), **C** and **D** total carbon (TC), **E** and **F** elemental carbon (EC), and **G** and **H** saturated isothermal remanent magnetisation (SIRM) volume concentration for France and Senegal samples

## Results

### Urban backgrounds

PM concentration in Senegal (39.1µg m$^{-3}$) is more than twice the mean PM in France (15.5µg m$^{-3}$) (Table 1). The PM concentration size distribution is unimodal in France (Fig. 1A) and bimodal in Senegal (Fig. 1B). In the two countries, PM concentration is mainly contained in the supermicron fraction ($D_{a}>{1}$µm) constituting 57% and 61% of the total PM concentration in France and Senegal, respectively. Mass geometrical mean diameter (GMD) is 1.0µm in France and Senegal.

**Fig. 2:**
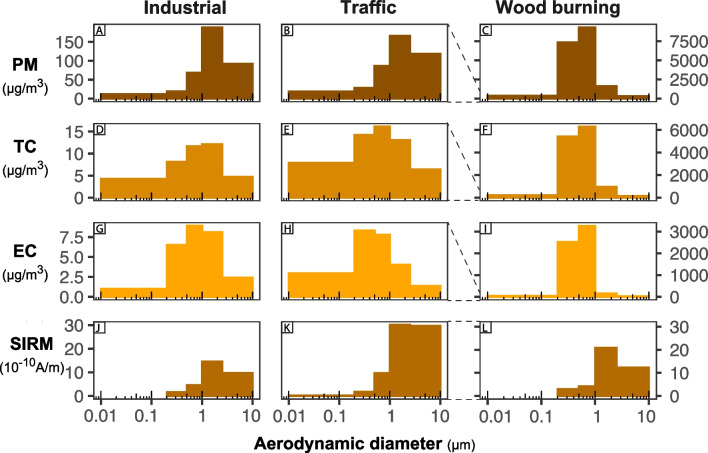
Size distribution by size fraction of **A**, **B**, **C** PM concentration; **D**, **E**, **F** TC concentration; **G**, **H**, **I** EC concentration; and **J**, **K**, **L** SIRM normalized by volume at **A**, **D**, **G**, **J** Senegal *industrial*; **B**, **E**, **H**, **K** Senegal *traffic*; and **C**, **F**, **I**, **L** Senegal *woodburning* sites. Modification of the *y*-axis for the Senegal *wood_burning* site

Total TC concentration is 2.1µg m$^{-3}$ and 5.4µg m$^{-3}$ in France and Senegal, respectively (Table 1). The concentration distribution of carbonaceous species (TC and EC) is unimodal (Fig. 1C, D, E, and F). The GMD for the size-fractionated EC and TC are similar in France and Senegal (EC = 0.7µm and 0.6µm respectively, TC = 0.6µm in both countries). In the two countries, the submicron fraction ($D_a<{1}$µm) contains the highest TC concentrations. Submicronic ($D_a<{1}$µm) TC is equal to 71% and 68% of the total TC concentration in France and Senegal, respectively. The submicron fraction ($D_a<{1}$µm) of EC represents 71% of the total EC concentration in France (total EC $= {0.6}$µg m$^{-3}$) and 75% in Senegal (total EC $= {1.6}$µg m$^{-3}$).

The OC/EC ratio for PM10 is 2.4 in Senegal and 2.3 in France. In both countries, the OC/EC ratio exhibits a bimodal distribution with a trough in the $0.2-{0.5}$µm fraction for both locations due to the prevalence of EC in the fine fraction ($D_a$ between 1 and 0.2µm). OC/EC is the highest in the coarse ($D_a>{2.5}$µm) and quasi-ultrafine ($D_a<{0.2}$µm) fraction for both sites.

**Fig. 3:**
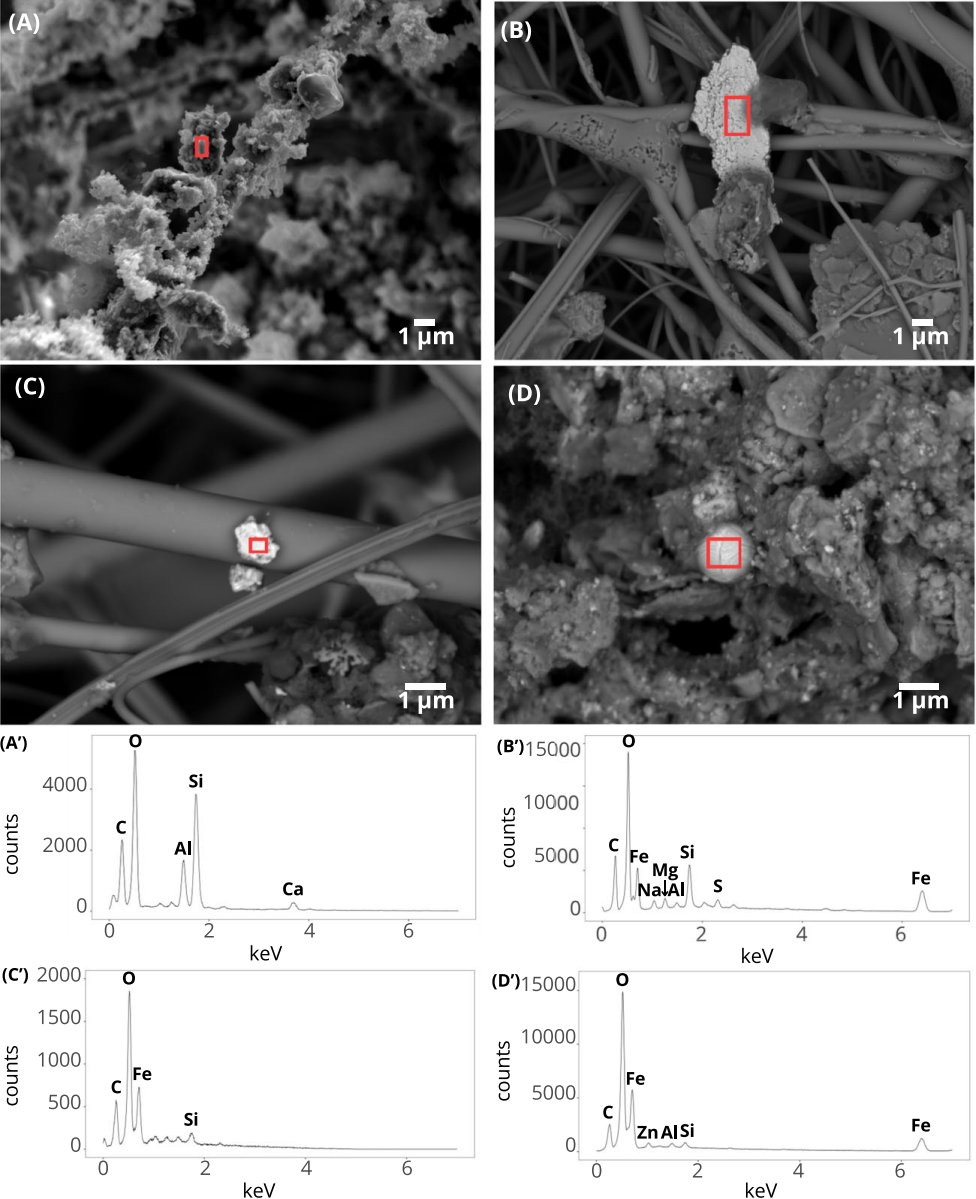
SEM image of **A** carbon fluffy soot aggregate (LED) collected at the urban background site in Senegal on stage 4 (0.2µm$<D_a<{0.5}$µm), **B** iron-rich particles (BED) collected at the urban background site in France on stage 1 ($D_a>{2.5}$µm), **C** on stage 2 (1µm$<D_a<{2.5}$µm) (BED), and **D** iron-rich particles (BED) collected at the urban background site in Senegal on stage 1 ($D_a>{2.5}$µm). (**A’** - 10kV), (**B’** - 10kV), (**C’** - 5kV), and (**D’** - 10kV) EDS spectrum of the particle in the respective red square of image (A), (B), (C), and (D)

**Fig. 4:**
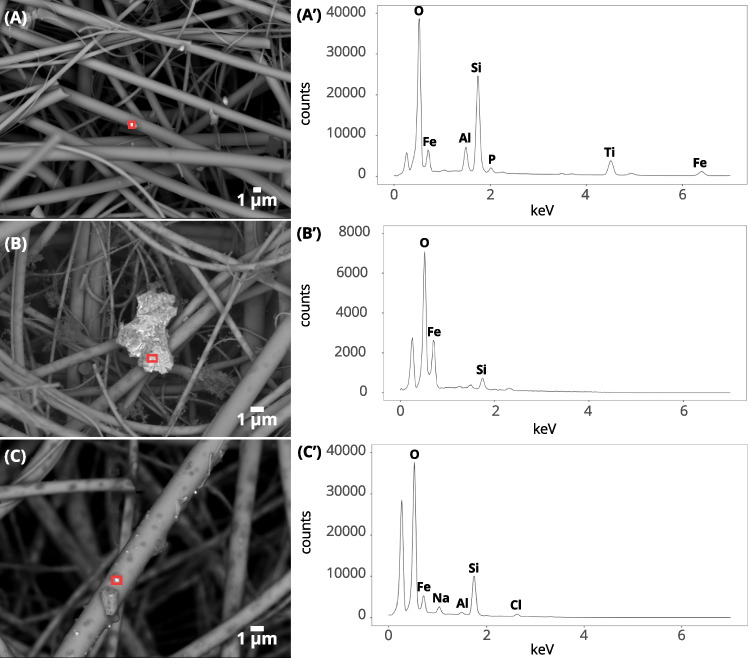
SEM images of iron-rich particles collected on the backup filter ($D_a<{0.2}$µm) at the **A** *industrial* site (BED), **B** *traffic* site (BED), and **C** *wood_burning* site (BED). (**A’** - 10kV), (**B’** - 5kV), and (**C’** - 5kV) EDS spectrum of the particle in the respective red square of image (A), (B), and (C)

**Fig. 5:**
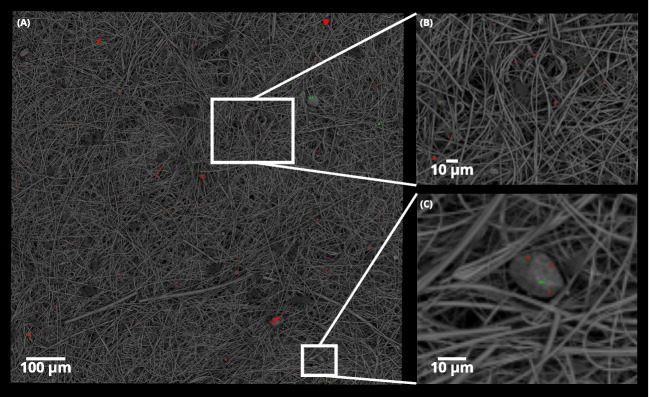
Detection of particles by EDS in the quasi-ultrafine fraction ($D_a<{0.2}$µm) on the backup filter of the *traffic* site in Senegal. Particles in red are iron-rich particles (Fe>5wt% or Fe wt%> Zn wt%) and particles in green are unclassified particles. **A** 1mm$^{2}$ area representing 252 fields of view. **B** and **C** Zoomed-in views. Scale bars are given for information only

The SIRM of Senegalese filters ($16.1\times 10^{-10} \text {A}\, \text {m}^{-1}$) is eight times higher than the one obtained on French filters ($1.9\times 10^{-10} \text {A}\, \text {m}^{-1}$) (Table 1). SIRM size-fractionated distributions are unimodal peaking in the supermicronic size fraction ($D_a>{1}$µm) (Fig. 1G and H). The supermicron fraction ($D_a>{1}$µm) has the highest SIRM, being 79% and 81% of total SIRM in France and Senegal, respectively. The GMD for the SIRM is 1.2µm and 1.4µm in France and Senegal, respectively. The SIRM mass ratio, that provides the magnetic content in PM in A m$^{2}$ kg$^{-1}$, varies between $0.6\times 10^{-2} \text {A}\, \text {m}^{2} \text {kg}^{-1}$ to $6.4\times 10^{-2} \text {A}\, \text {m}^{2} \text {kg}^{-1}$ (Table 1). The SIRM mass ratio is three times higher in Senegal than in France considering the whole size range and all size fractions (Table 1).

### Traffic, industrial, and wood burning sources in Senegal

To characterize specific sources in Senegal, we investigated 3 “sources” sites: a kerbside site (*traffic*), a secondary traffic site close to the steel factory (*industrial*), and inside a traditional barbecue shop (*wood_burning*).

Total PM concentrations are 4.4, 5.1, and 179.0 times higher at the *industrial* (173.1µg m$^{-3}$), *traffic* (198.6µg m$^{-3}$), and *wood_burning* (6997.0µg m$^{-3}$) sites than at the urban background site, respectively (Table 1). The PM concentration distribution is unimodal, with a maximum in the $1-{2.5}$µm size fraction at the *industrial* (Fig. 2A) and *traffic* (Fig. 2B) sites. The maximum PM concentration at the *wood_burning* site (Fig. 2C) is shifted towards smaller particles, with a maximum in the $0.5-{1.0}$µm fraction, resulting in a shift of the GMD toward lower size. GMD are 0.7µm, 1.1µm, and 1.2µm for the *wood_burning*, *traffic*, and *industrial* sites, respectively. PM concentration is mainly contained in the supermicron fraction ($D_a>{1}$µm) constituting 75% and 69% of the total PM concentration at the *industrial* and *traffic* sites, respectively. In contrast, at the *wood_burning* site the PM concentration is mainly contained in the $0.2-{1}$µm fraction which contains 81% of the total PM concentration.

Total TC concentrations are respectively 3.7, 5.6, and 893.8 times higher than the urban background site at *industrial* (20.0µg m$^{-3}$), *traffic* (29.8µg m$^{-3}$), and *wood_burning* (4782.9µg m$^{-3}$) sites, respectively (Table 1). The TC size distribution is unimodal with a maximum in the $0.5-{1}$µm fraction at the *traffic* (Fig. 2E) and *wood_burning* (Fig. 2F) sites and with a maximum in the $1-{2.5}$µm fraction at the *industrial* (Fig. 2D). The TC concentration is driven by particles smaller than 0.2µm at *industrial* and *traffic* sites. The submicron ($D_a<{1}$µm) percentage of TC is 61%, 70%, and 90% for the *industrial*, *traffic*, and *wood_burning*, respectively. EC has a similar size distribution than TC (Fig. 2G, H, and I). The highest OC/EC ratio is 5.8 and observed at the *wood_burning* for particles above 2.5µm indicating large OC emission due to the biomass combustion. The lowest OC/EC for PM10 is 0.8 and observed at the *industrial* site located close to the main traffic road.

The SIRM of sources filters is $12.5 \times 10^{-10} \text {A}\, \text {m}^{-1}, 33.6 \times 10^{-10} \text {A}\, \text {m}^{-1}$, and $17.0\times 10^{-10} \text {A}\, \text {m}^{-1}$ (Table 1) at the *industrial*, *traffic*, and *wood_burning* which is 1 and 2 times higher than the SIRM at the urban background site, respectively. No significative values could have been obtained for the backup filter for the *wood_burning* and *industrial*. The size-fractionated SIRM distributions are unimodal. Their maximum is in the $1-{2.5}$µm size fraction for *industrial* (Fig. 2J) and *wood_burning* (Fig. 2K) sites and in the $1-{10}$µm for the *traffic* site (Fig. 2L). Similarly to the urban background site, the SIRM of the submicron fraction ($D_a<{1}$µm) represents 85%, 87%, and 89% of the total SIRM for the *industrial*, *wood_burning*, and *traffic* sites, respectively.

The SIRM mass ratio is highest at the *traffic* site, regardless of the size fraction (Table 1). However, SIRM mass ratio of all sources is lower than those at the background urban site whatever the size fraction is.

### SEM particles detection and characterization

To identify the presence, the shape of magnetic particles (iron-rich particles) and their association with carbonaceous particles, we performed SEM observations and EDS analysis on all filters collected at the two urban sites and all the backup filters at Senegal sources sites.

In France and Senegal, we observed carbon-rich chains associated with iron-rich particles and sometimes zinc-rich ones. Fluffy soot aggregates with carbon-rich chains (Fig. 3A and A’) are observed at the two urban sites in France and Senegal. Iron-rich particles, irregular flakes and spherules, are visible at all sites (Fig. 3B, B’, C, and C’). Irregular iron-rich particles are more abundant than spherules. They have sharp edges and could have a smooth or rough surface. Spherical iron-rich particles have mainly been found in samples from Senegal urban background site (Fig. 3D and D’) trapped on soot aggregates. Traces of other elements, such as Cu, Sn, Pb, and Ba in France and Ba, Cu, and Pb in Senegal, are detected alongside with carbon particles and iron-rich particles, notably in particles smaller than 1 µm in size at the Senegal urban site. We noticed that certain particles exhibited a diameter larger than expected for the given stage (Fig. 4B), likely to be associated with contamination of the stage by a rebound effect.

Iron-rich particles were detected in all size fractions, even in the quasi-ultrafine fraction ($D_a<{0.2}$µm), although they were not detected using magnetic property analysis techniques (Fig. 4A, A’, B, B’, C, and C’). Quasi-ultrafine iron-rich particles ($D_a<{0.2}$µm) are harder to detect, as they move under the influence of electrons during the scan.

To facilitate their detection and identification, we performed EDS mapping of 1mm$^{2}$ (Fig. 5A, B, and C). We identified 551 iron-rich particles (Table A2) in the 1mm$^{2}$ aliquot for the *traffic* site. The iron-rich particles had an average equivalent circle diameter (ECD) of 0.79µm. The finest particles appeared to be spherical. For the *industrial* site, 476 iron-rich particles (with a mean ECD of 0.68µm) and 3 zinc-rich particles were detected (Table A2). The *wood_burning* site showed less iron-bearing particles (327) with a mean ECD of 0.42µm (Table A2).

### Rock magnetism: hysteresis parameters

All hysteresis curves (Fig. A1) are quite narrow with coercivities (Hc) between 11.7mT and 20.7mT for the Senegalese sites and with slightly lower coercivities (8.4–10.6mT) for the French background site. The curve of the first stage ($D_a>{2.5}$µm) at the *industrial* site shows a slightly wasp waisted shape (Fig. A1B). Wasp-waisted hysteresis curves (Tauxe et al. 1996) could represent either a combination of large superparamagnetic (SP) particles and single domain (SD) magnetite or a mixture of different coercivities. Given the size range considered and a possible lithic influence, the wasp-waisted shape is likely to represent a mixture of hematite/goethite and magnetite-like grains.

**Fig. 6:**
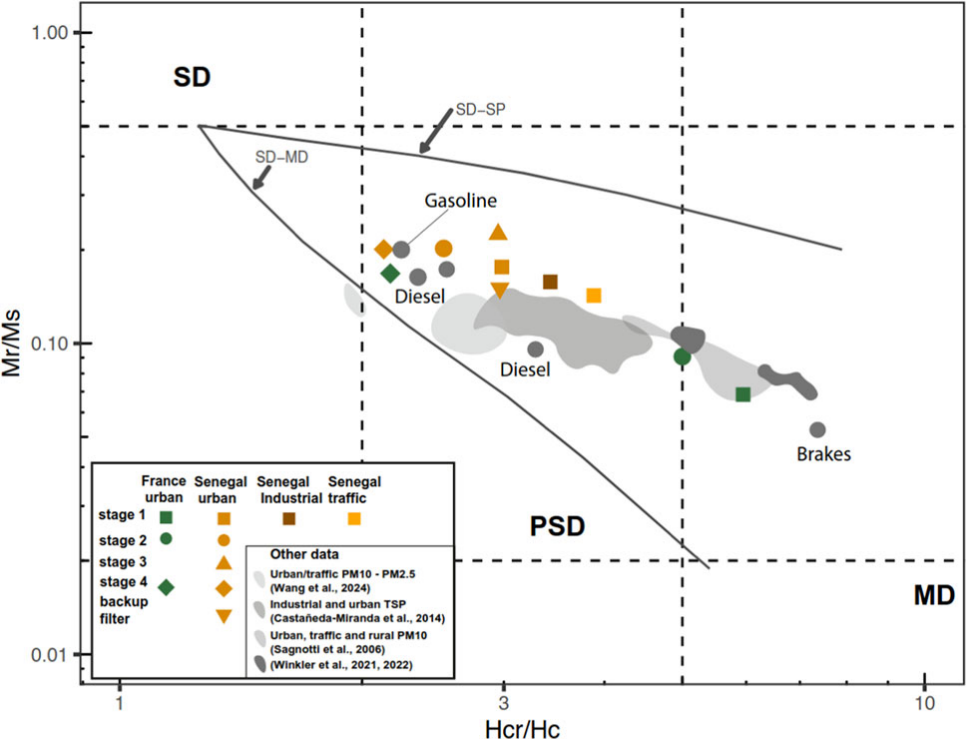
Bilogarithm Day plot (Dunlop 2002a) displaying hysteresis parameters of (orange) stage 1, 2, 3, and 4 and backup filters collected in Senegal urban background; (brown) of stage 1 at the *industrial* site, (yellow) at the *traffic* site and (green) of stage 1, 2, and 4 at the France urban background; (gray) additional data from PM10 and PM2.5 at an urban/traffic site in Lanzhou City, China Wang et al. (2024); Total Suspended Particulate (TSP) at industrial and city center (urban) sites in Santiago de Querétaro, Mexico Castañeda-Miranda et al. (2014); PM10 at urban, traffic and rural sites in Latium territory, Italy Sagnotti et al. (2006); PM10 at traffic and rural sites in Rome, Italy Winkler et al. (2021) and traffic exhaust and non-exhaust emissions in Italy Winkler et al. (2022). Single domain (SD), pseudo-single domain (PSD), and multi-domain (MD) along with mixing curves (SD-MD) and (SD-SP) are from Dunlop (2002a, 2002b)

The hysteresis parameters (Fig. 6) (Mrs/Ms and Hcr/Hc) of all the Senegalese samples revealed that all the particles belonged to the theoretical pseudo-single domain (PSD) area of the Day plot (Dunlop 2002a; Day et al. 1977). Samples from Senegal urban background fall more slightly towards the SD domain range than the *industrial* and *traffic* samples. In contrast, the parameters of the first two stages ($D_a>{2.5}$µm and 1µm$<D_a<{2.5}$µm) of France urban background site fall between the multidomain (MD) and SD theoretical areas of the Day plot (Fig. 6). However, the fourth stage data ($D_a$ between 0.5 and 0.2µm) lies close to the Senegal urban background site near the SD limit of the PSD area.

**Fig. 7:**
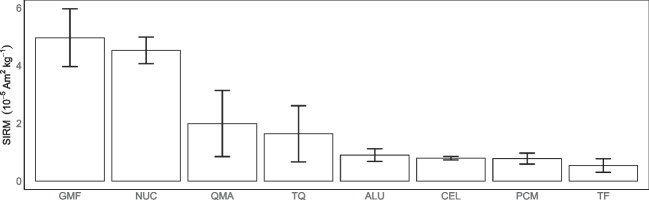
SIRM mass ratio for 8 clean (before exposure) sampling media: Fiberglass (GMF), nucleopore (NUC), Whatman quartz (QMA), quartz fiber filter (TQ), aluminum (ALU), cellulose ester (CEL), polycarbonate membrane (PCM), and Teflon (TF). Error bars are the standard deviations

### Influence of types of filter on magnetic signal

All the samples presented here were collected on quartz fiber filters grade QMA. To investigate the impact of the choice of the sampling media on SIRM, we selected different types of media commonly used in aerosol research. A total of 8 media, aluminum (ALU), cellulose ester (CEL), fiberglass (GMF), nucleopore (NUC), polycarbonate membrane (PCM), Whatman quartz (QMA), Teflon (TF), and quartz (TQ) were analyzed. The average of the 5 SIRM measurements per type of media is presented in Fig. 7. Filter media present a large range of mass normalized SIRM from $(5.4 \pm 2.4) \times 10^{-6} \text {A}\, \text {m}^{2} \text {kg}^{-1}$ to $(49.7 \pm 10.0) \times 10^{-6} \text {A}\, \text {m}^{2} \text {kg}^{-1}$ in the following order GMF > NUC > QMA > TQ > ALU> CEL > PCM > TF. ALU, CEL, PCM, and TF have a rather low SIRM mass ratio and low standard deviation.

The influence of sampling media on magnetic properties is a novel observation in this study. Quartz fiber filters grade QMA are generally used in aerosol sampling studies, as they enable both chemical and magnetic analysis. Here, we demonstrated that the choice of the sampling media has a major impact on the interpretation of magnetic data.

## Discussion

PM emissions from combustion and non-combustion processes are intrinsically mixed in the atmosphere. Our study provides a new insight into the mixture of the two types of emissions by jointly analyzing the magnetic signature and carbon content throughout the particle size distribution.

### Characteristic of Western African urban sources and environments

#### Wood burning site

To identify the signature of the wood-burning source, we sampled particles in a wood-fired cooking street restaurant at the *wood_burning* site. We collected a high PM concentration of 6997.0µg m$^{-3}$ in three hours. The *wood_burning* displays specific characteristics. The narrow PM size distribution is concentrated mainly in the $0.2-{1}$µm size fraction. PM contains a high carbonaceous aerosol content, as revealed by the high concentrations of TC and EC in the same fractions ($0.2-{1}$µm). However, the concentration of magnetic particles (represented by SIRM by air volume) does not follow this pattern. Instead, it exhibits high values in the first stages. High values of SIRM by air volume have led to an SIRM mass ratio of $0.4 \times 10^{-2} \text {A}\, \text {m}^{2} \text {kg}^{-1}$ and $0.1 \times 10^{-2} \text {A}\, \text {m}^{2} \text {kg}^{-1}$ for the $2.5-{10}$µm and the $1-{2.5}$µm size fractions, respectively. The SIRM indicates the presence of magnetic particles in the $1-{10}$µm stages. However, the low SIRM mass ratio values indicate that wood burning does not contribute significantly to the emission of magnetic particles. The *wood_burning* site is a street food store and, as such, is largely contaminated by emissions from road traffic.

High concentration of particles in the $0.1-{0.2}$µm fraction is characteristic of the frequent use of household biofuels due to the high contribution of carbonaceous aerosols in the fine fraction of biomass combustion (Kleeman et al. 1999). Wood burning is known to massively emit organic species (Liousse et al. 2010; Torvela et al. 2014). OC/EC ratios between 5.2 and 6.0 have been reported for a meat-smoking area in Abidjan (Djossou et al. 2018; Leite et al. 2021). At the wood-burning site in Senegal, the emission of OC is reflected by a high OC/EC ratio of 5.3 and 5.8 for particles above 1µm. However, the OC/EC ratios calculated for equivalent PM2.5 and PM10 are 1.2 and 1.3 respectively, as they include stages smaller than 1 micron, which have the highest TC concentrations and ratios below 1.1. The lower values reported here for the *wood_burning* site could be due to flaming rather than smoldering conditions.

The association between PM mass concentration and the concentration of magnetic particles is not always straightforward, and even negative correlations have been reported (Petrovský et al. 2020; Winkler et al. 2021). In this case, we note that the intense emission of carbonaceous particles by wood burning is not associated with a significant increase in the magnetic signal whatever the size fraction in line with previous studies (Leite et al. 2021).

#### Traffic site

The traffic site in Sebikotane allowed us to collect particles from wear and abrasion of circulating vehicles in the national road, as well as emissions from exhaust. In addition, the sampling includes particles from resuspension, consisting of a mix between traffic-related particles and natural dust coming from arid areas. The PM size concentration distribution peaks in the coarsest stages and is distributed in the range of size from 1 to 10µm. Similar PM concentration size distribution has been found in Braga (Portugal) in a road tunnel (Alves et al. 2015), Vellore city (Tamil Nadu state of southern India) at a roundabout nearer a city bus station (Manojkumar and Srimuruganandam 2022), and Dunkirk region (France) downwind of the urban and traffic emissions from the highways (Mbengue et al. 2014). The PM concentrations (PM10$={198.6}$µg m$^{-3}$ and PM2.5$={127.2}$µg m$^{-3}$) and the TC concentrations (PM10$={29.8}$µg m$^{-3}$ and PM2.5$={26.0}$µg m$^{-3}$) are of the same order as those reported for heavy traffic in Dakar (PM concentrations of: PM10$={155.9}$µg m$^{-3}$ and PM2.5$={138.2}$µg m$^{-3}$; TC concentrations: PM10$={51.3}$µg m$^{-3}$ and PM2.5$={51.7}$µg m$^{-3}$) (Doumbia et al. 2023). EC content can be used as a reliable direct indicator of exhaust emissions; the OC/EC ratio can help specify combustion sources. An OC/EC ratio close to 2 indicates a mixture of contributions from old diesel (OC/EC ratio <1) and petrol (OC/EC ratio >1) combustion engine (Alves et al. 2015). At the traffic site, the OC/EC for the equivalent PM2.5 is 1.3 in the same order as those reported for Abidjan (between 1.0 and 3.0), Cotonou (between 2.5 and 4.5) and Dakar (2.3) (Djossou et al. 2018; Doumbia et al. 2023), indicating the influence of diesel traffic at the traffic site in this study. Here, this low OC/EC ratio is accounted for by the predominance of diesel vehicles in Sebikotane’s traffic on the national highway.

Non-exhaust emissions from traffic have recently been reported in the UK to release magnetic minerals in the coarse size fraction corresponding to the $2-{10}$µm fraction (Beddows et al. 2023). Road traffic contributes to magnetic mineral emissions through the wear of brake pads and tyres (Fussell et al. 2022; Beddows et al. 2023). Wahlström et al. (2010); Hussain et al. (2014). Nevertheless, road traffic is known to also emit magnetic spherules during the combustion processes (Leite et al. 2021). Indeed, such iron-rich spherules have been identified by SEM observation. However, iron-rich spherules do not appear to contribute significantly to the SIRM signal as the $1-{10}$µm stages are dominated by irregular iron-rich particles (Fig 3). Spherules influence the hysteresis parameters by shifting the values toward the “exhaust” pole (Fig 6) (Sagnotti et al. 2009; Winkler et al. 2022).

#### Industrial site

Two steel-recycling industries may contribute to the emission of iron-rich particles in the urban area of Sebikotane. The steel transformation industry emits magnetic minerals (Dall’Osto et al. 2008). The *industrial* site was chosen as close as possible to the steel-recycling industry, access to which is restricted. However, the site where the sampler was positioned was 50µm from the main road, and the light wind did not carry emissions from the industry towards the chosen house during the sampling period. We chose to sample during the night and in the morning until midday, to avoid as far as possible the influence of traffic, which is reduced during the night. As a result, the *industrial* site is influenced by road traffic and slightly by emissions from the steel industry. However, few zinc-rich particles were found in the backup filter (3 particles classified as zinc-rich particles (Table A2)). Zinc could be released by traffic (Pant and Harrison 2013; Garg et al. 2000) or by the steel industry (Querol et al. 2007; Zhou et al. 2014). As no zinc-rich particles were identified in the sampling at the traffic site, we assume that the zinc-rich particles originate from industry activities. In addition, we identified more iron-rich particles (476 particles classified as iron-rich particles at industrial in 1mm$^{2}$ (corresponding to a duration of 5 h of traffic activity considering that traffic begins at 7 h)) compared to the traffic site (551 iron-rich particles at traffic in 1mm$^{2}$ (14 h 35’ of traffic activity)) (Table A2). SEM results indicate that the industrial source emits zinc- and iron-rich particles in the quasi-ultrafine fraction ($Da<{0.2}$µm), although the sampling of industrial emissions is not optimal and they are mixed with the influence of traffic. The iron-rich contribution is not reflected in the magnetic signal (SIRM), which remains weaker than in the *traffic* site. However, the hysteresis loop (Fig. A1) of the first stage reflects a possible input of high-coercivity minerals that could come either from natural dust (Lyons et al. 2010; Formenti et al. 2014), or from specific industrial emissions.

The PM size concentration distribution at the industrial site reaches a maximum in the $1-{2.5}$µm size fraction. Similar PM concentration size distribution has been found at the traffic site but with a lower contribution from the $2.5-{10}$µm size fraction at the *industrial* site than at the *traffic* site. The two size distributions of PM concentration show similar patterns, probably linked to the influence of traffic emissions due to the proximity of the road to the industrial sampling site. At the *industrial* site, the OC/EC ratio for the equivalent PM2.5 is 0.7 with the prevalence of EC in the carbonaceous aerosols emissions. An OC/EC ratio of 0.7 cannot only be associated with an industrial influence due to the proximity of the road, despite the fact that the OC/EC ratio at the *industrial* site is lower than that identified at the *traffic* site.

#### Sources identification at the urban background Senegalese site

The urban background site is located 850m south of the steel-recycling industry and is therefore exposed to the general contribution of anthropogenic activities — traffic, industry, biomass combustion — and to the contribution of wind-borne lithic dust. The longer sampling period (48 h, Table A1) at the urban background site than at the other sites in Senegal means that all these sources are likely to have been sampled. The number of iron-rich particles (1944 in 1mm$^{2}$ (Table A2)) identified in the backup filter at the urban background site is three times higher than at the traffic site. Sampling at the urban background site was conducted during 48 h (Table A1) including 30 h during work hours with traffic intense activities which is twice the sampling period at the traffic site (14 h 35’, Table A1). Similarly, the comparatively higher number of zinc-rich particles at the urban background site (353 in 1mm$^{2}$ (Table A2)) than at the traffic site (0 in 1mm$^{2}$ (Table A2)) reveals the influence of the industrial source of zinc. The industrial influence is clearly marked by the identification of 3 zinc-rich particles (Table A2) in the industrial site’s backup filter for a sampling period of 12 h 55’ (Table A1), including only 5 h during intense road traffic.

Senegalese sites are characterized by the presence of carbon particles in the quasi-ultrafine fractions except in the case of the wood-fired cooking street restaurant where they are emitted in the $0.2-{0.5}$µm size range. Magnetic particles express their presence in the coarse fractions of all sources and at the urban background site. However, iron-rich particles are also present in the quasi-ultrafine stage at all sites.

### Comparing the urban environment in Senegal and France

We found significant differences between sampling in urban areas in France and Senegal. The PM size concentration distribution in France is unimodal and reaches a maximum in the $1-{2.5}$µm size fraction. In Senegal, the PM size concentration distribution is bimodal peaking in the $<{0.2}$µm and $1-{2.5}$µm size fractions. The PM concentration and the TC content in the urban background in Senegal were more than twice those recorded in France. PM concentrations are comparable to those generally reported for Western Africa and Europe, respectively. For instance, urban background PM10 (15.5µg m$^{-3}$) and PM2.5 (11.2µg m$^{-3}$) mass concentrations data recorded here in France are comparable to those (PM10 = 15.3 to 17.6µg m$^{-3}$ and PM2.5 $= 8.9$ to 14.8µg m$^{-3}$) reported at four air monitoring sites at Valladolid (Spain) (e.g., García et al. 2019). The instantaneous PM2.5 concentration of 25.5µg m$^{-3}$ at the urban background in the commune of Sebikotane is the same order of magnitude as the annual and biennial PM2.5 concentration estimated in western African countries, as in the case of the economic capital of Côte d’Ivoire, Abidjan with PM2.5 concentrations of 16.5 to 29.6µg m$^{-3}$ (Gnamien et al. 2023; Bahino et al. 2024).

Total OC/EC ratios are similar for the urban background in France (PM10: OC/EC=2.3) and Senegal (PM10: OC/EC=2.4). Carbonaceous particles (soot) derive from combustion processes. We found that OC/EC depends on particle aerodynamic diameter with the highest values in the coarse and quasi-ultrafine fractions. The contribution of OC increased in the quasi-ultrafine fraction indicating the possible contribution of secondary aerosols (Mirante et al. 2014). Difference in the OC/EC ratio in the quasi-ultrafine fraction could indicate a contribution from biomass burning as OC concentration is significantly higher in the quasi-ultrafine fraction in Senegal than in France and/or a difference in the traffic-related emissions with higher EC aerosols emit by old combustion engine (Senegal) in the quasi-ultrafine fraction than by recent combustion engine (France). The SIRM mass ratio for the coarse fraction of the Senegal urban background is much higher than in France and even higher than the ones at the *industrial* and *traffic* sites. The SIRM mass ratio at the background environment in Senegal for the quasi-ultrafine fraction of $1.8 \times 10^{-2} \text {A}\, \text {m}^{2} \text {kg}^{-1}$ is very close to the values (SIRM mass ratio = $2.23 \times 10^{-2}$ and $2.28 \times 10^{-2} \text {A}\, \text {m}^{2} \text {kg}^{-1}$) found by Leite et al. (2021) for PM2.5 in Abidjan (Côte d’Ivoire) and Cotonou (Bénin). The difference in the SIRM mass ratio between France and Senegal in the coarse fraction could be explained by the composition of the vehicle fleet and probably by the contribution of lithic particles.

### Non-combustion versus combustion-related proxies: magnetic and carbon signal

The relative importance of carbonaceous aerosols and magnetic minerals differs according to the aerodynamic diameter of aerosols, with a greater proportion of carbonaceous particles in the finest fractions and a predominance of magnetic particles in the coarsest stages. However, the two particle types appear to be closely associated in the observations. Indeed, carbonaceous particles appear as fluffy soot aggregates associated with magnetic minerals (iron-rich particles).

Magnetic minerals exhibit different morphologies, including spherical and irregular shapes, depicting various emission processes combustion, and non-combustion related. Iron-rich spherules are associated with combustion emissions (Flament et al. 2008; Ault et al. 2012; Gonet and Maher 2019; Liati et al. 2013). The analysis of the magnetic parameters revealed the presence of low-coercivity magnetic minerals across all samples. The low-coercivity values (Larrasoaña et al. 2021; Xiao et al. 2022) indicate the dominance of soft ferromagnetic materials associated with anthropogenic emissions, magnetite or maghemite (Stachurski et al. 2021). Magnetic hysteresis parameters clearly show different magnetic signatures for urban environments in Senegal and France. We can conclude that magnetic minerals in Senegal and France originate from a mixture of iron-rich PM10 and PM2.5 particles and that the finest iron-rich particles are combustion-related. In France, hysteresis parameters indicate that the urban background site is largely influenced by non-exhaust traffic-related supermicron particle emissions and exhaust traffic-related submicron particle emissions.

One limitation of our approach is that it is particularly difficult to accumulate mass on the finer fractions of the cascade impactor, since it is the coarser fractions that contain the most mass. Increasing the sampling duration would enable greater mass to be accumulated on the finer fractions. However, it would also have the effect of overloading the coarser fractions in the impactor which could lead to contamination of lower levels by the rebound effect of particulate matter. The rebound effect was observed in the *traffic* backup filter (Fig. 5). The low accumulated mass has led to weak SIRM signals that are close to the clean filter ones. Indeed, the sampling media plays an important role in the background noise level of the magnetic signal. We demonstrated that QMA filters have a high SIRM and a large variability from one filter to the other. In this study, the choice of QMA filters for our samplings was motivated by the possibility of carrying out thermo-optical and magnetic analyses on the same medium.

Finally, superparamagnetic particles can be difficult to detect using SIRM measurement due to the fact that they cannot keep their magnetization. To evaluate the presence of superparamagnetic particles, the use of frequency-dependent magnetic susceptibility or low-temperature and high-field measurements would be a further step in the investigation of particles $<{0.2}$µm, but the low magnetic signal of the finest fraction could hamper this.

## Conclusion

The size distribution of PM concentrations, carbonaceous fraction (TC), and magnetic mineral concentration (SIRM) were investigated using aerosol size segregation carried out in two very different urban environments in France (Toulouse) and Senegal (Sebikotane). We can conclude that:

- The PM concentration in urban environments in Senegal is twice as high as the mean PM concentration in France. PM mass size distribution in France is unimodal, whereas in Senegal it is bimodal. The geometric mean mass diameter (GMD) is 1.0µm in both countries;
- Carbonaceous aerosols are mainly concentrated in the submicron fraction, with more than 70% of EC being in the submicron fraction;
- The OC/EC ratio is similar for both urban backgrounds (around 2.3) and exhibits a bimodal distribution, with the highest values observed in the coarsest and the finest fractions;
- Wood burning is linked to intense emission of carbonaceous particles which is not associated with a significant magnetic signal. As the measurements were carried out on a street food site, it is likely that the magnetic fraction of the samples are also largely influenced by road traffic;
- Approximately 80% of the magnetic signal is contained within the supermicron fraction in France and Senegal;
- We note that the choice of the sampling media (filter) has an impact on the magnetic measurements. Filter media SIRM ranges between $(5.4\pm 2.4)\times 10^{-6} \text {A}\, \text {m}^{2} \text {kg}^{-1}$ and $(49.7 \pm 10.0) \times 10^{-6} \text {A}\, \text {m}^{2} \text {kg}^{-1}$;
- The difference in the particle size distribution of the carbonaceous aerosols and magnetic minerals concentrations reveals different emission mechanisms, between exhaust (carbonaceous aerosols and magnetic minerals) and non-exhaust (magnetic minerals);
- The concentration of magnetic minerals per mass in the urban background of the medium-sized city in Senegal is three times higher than that observed in the large city in France. While this result is specific to the case studies presented in this paper, it likely reflects the combined contributions of industrial emissions and natural terrigenous aerosols to airborne magnetic particle levels.

## Supplementary Information

Below is the link to the electronic supplementary material.

Supplementary file 1 (pdf 236 KB)

## Data Availability

The authors declare that the data supporting the findings of this study are available within the paper, its supplementary information files.
