# Supplementary material for: Size-fractionated carbonaceous and iron-rich particulate matter in urban environments of France and Senegal
Source: Environ Sci Pollut Res Int. 2024 Dec 19;32(2):677–92. doi: 10.1007/s11356-024-35729-x (PMC11732942; doi:10.1007/s11356-024-35729-x)
Supplement: Supplementary file 1 — (pdf 236 KB) [file 11356_2024_35729_MOESM1_ESM.pdf]

Size-fractionated carbonaceous and iron-rich particulate matter in urban environments of France and Senegal.

Laurence Delville[1,2*], Jean-François Léon[1], Mélina Macouin[2], Yann-Philippe Tastevin[3], François Demory[4], Arnaud Proietti[5], Pedro Henrique da Silva Chibane[6], Maria Dias Alves[1], Mayoro Gueye[3], Laure Laffont[2], Eric Gardrat[1], Sonia Rousse[2], Loic Drigo[2], Andréa Teixeira Ustra[6]

[1*]Laboratoire d'Aérologie, Université de Toulouse, CNRS, IRD, UPS, Toulouse, France.

[2]Géosciences Environnement Toulouse, Université de Toulouse, CNES, CNRS, IRD, UPS, Toulouse, France.

[3]OHMi Tessekere, ESS - Unité Mixte Internationale "Environnement Santé Sociétés", Université Cheikh Anta Diop de Dakar, Dakar, Sénégal.

[4]Centre Européen de Recherche et d'Enseignement des Géosciences de l'Environnement, Aix-Marseille Université, CNRS, IRD, INRAE, Aix-en-Provence, France.

[5]Centre de micro-caractérisation Raimond Castaing, Université de Toulouse, CNRS, INP Toulouse, INSA Toulouse, UFTMP, UPS, Toulouse, France.

[6]Instituto de Astronomia, Geofísica e Ciências Atmosféricas, Universidade de São Paulo, USP, São Paulo, Brazil.

[*]Corresponding author: Laurence Delville, laurence.delville@aero.obs-mip.fr, +33613593832.

Journal : Environmental Science and Pollution Research.

Appendix A Supplementary material

Table A1 Duration of particle sampling by cascade impactor in France (urban background) in 2022 and in Senegal in February 2023

France urban		Senegal	
April	95h 15'	Urban	48h
May	48h 48'	Industrial	12h 55'
October	69h 58'	Traffic	14h 35'
November	113h 36'	Wood burning	3h

Table A2 Number of iron- and zinc-rich particles over a 1 mm^2 area in the backup filter (D_a <0.2 µm) for the samples collected in France and in Senegal

	Number of iron-rich particles	Number of zinc-rich particles
France $_{urban}$	65	0
Senegal $_{urban}$	1944	353
Senegal $_{industrial}$	476	3
Senegal $_{traffic}$	551	0
Senegal $_{wood\ burning}$	327	0

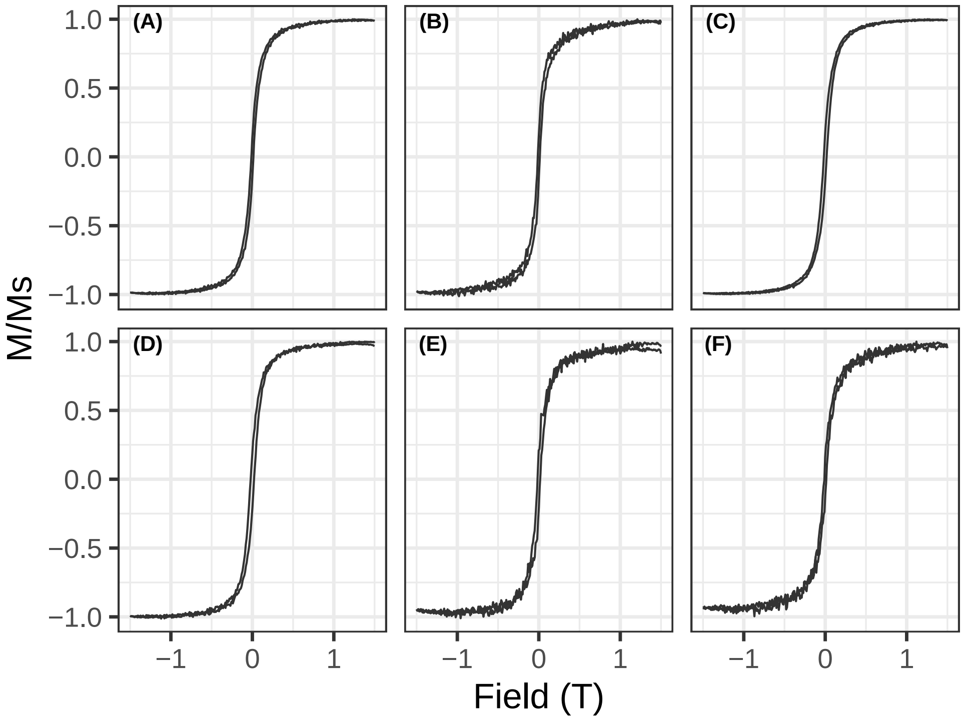

Fig. A1 Hysteresis loop for particles collected in Senegal on the first stage (D_a >2.5 µm) at the (A) *traffic*, (B) *industrial* and (C) urban background site on the (D) second stage (1 µm< D_a <2.5 µm), (E) third stage (0.5 µm< D_a <1 µm) and (F) backup filter (D_a <0.2 µm) at the urban background site.
